# Deimplementation in the provision of opioid agonist treatment to achieve equity of care for people engaged in treatment: a qualitative study

**DOI:** 10.1186/s13012-023-01281-4

**Published:** 2023-06-09

**Authors:** Anna Conway, Alison D. Marshall, Sione Crawford, Jeremy Hayllar, Jason Grebely, Carla Treloar

**Affiliations:** 1 The Kirby Institute, UNSW, Sydney, Australia; 2 Centre for Social Research in Health, UNSW, Sydney, Australia; 3 Harm Reduction Victoria, Melbourne, Australia; 4 Alcohol and Drug Service, Metro North Mental Health, Metro North Hospital and Health Service, Brisbane, Australia

**Keywords:** Drug treatment, Normalisation process theory, People who inject drugs, Methadone, Buprenorphine

## Abstract

**Background:**

Deimplementation, the removal or reduction of potentially hazardous approaches to care, is key to progressing social equity in health. While the benefits of opioid agonist treatment (OAT) are well-evidenced, wide variability in the provision of treatment attenuates positive outcomes. During the COVID-19 pandemic, OAT services deimplemented aspects of provision which had long been central to treatment in Australia; supervised dosing, urine drug screening, and frequent in-person attendance for review. This analysis explored how providers considered social inequity in health of patients in the deimplementation of restrictive OAT provision during the COVID-19 pandemic.

**Methods:**

Between August and December 2020, semi-structured interviews were conducted with 29 OAT providers in Australia. Codes relating to the social determinants of client retention in OAT were clustered according to how providers considered deimplementation in relation to social inequities. Normalisation Process Theory was then used to analyse the clusters in relation to how providers understood their work during the COVID-19 pandemic as responding to systemic issues that condition OAT access.

**Results:**

We explored four overarching themes based on constructs from Normalisation Process Theory: adaptive execution, cognitive participation, normative restructuring, and sustainment. Accounts of adaptive execution demonstrated tensions between providers’ conceptions of equity and patient autonomy. Cognitive participation and normative restructuring were integral to the workability of rapid and drastic changes within the OAT services. Key transformative actors included communities of practice and “thought leaders” who had long supported deimplementation for more humane care. At this early stage of the pandemic, providers had already begun to consider how this period could inform sustainment of deimplementation. When considering a future, post-pandemic period, several providers expressed discomfort at operating with “evidence-enough” and called for narrowly defined types of data on adverse events (e.g. overdose) and expert consensus on takeaway doses.

**Conclusions:**

The possibilities for achieving social equity in health are limited by the divergent treatment goals of providers and people receiving OAT. Sustained and equitable deimplementation of obtrusive aspects of OAT provision require co-created treatment goals, patient-centred monitoring and evaluation, and access to a supportive community of practice for providers.

Contributions to the literature

- The removal of potentially hazardous approaches to healthcare is key to achieving social equity in health, yet deimplementation processes can risk widening inequities
- This analysis demonstrates that constructs drawn from Normalisation Process Theory (adaptive execution, cognitive participation, normative restructuring, and sustainment) are useful to explore deimplementation work to understand how it can produce more equitable outcomes
- The findings have implications for opioid agonist treatment programs which have changed drastically across the world since the beginning of the COVID-19 pandemic, but also for other settings where rapid deimplementation risks entrenching social inequities in health

## Background

People who use illicit drugs are vulnerable to an unregulated drug market and lack of safe supply [1]. Opioid agonist treatment (OAT) can mitigate the harms associated with illicit opioids by reducing injecting risk behaviour, risk of HIV infection [2] and risk of hepatitis C infection [3]. OAT is also associated with reductions in all-cause mortality and drug-related mortality [4]. OAT is often tightly controlled with requirements for treatment including supervised dosing, urine drug screening, and frequent, in-person attendance for review [5]. Systematic reviews have attempted to assess the impact of urine drug screening [6] and supervised dosing [7] on various health outcomes, but these efforts have been hampered by a lack of studies. Restrictions on takeaways burden people by conditioning their daily routine and preventing feelings of “normality” [8], as well as presenting a barrier to adherence [9].

In Australia, there are significant variations in OAT provision between jurisdictions due to decentralised funding of health services and the varied historical contexts across the country [10]. In some jurisdictions, OAT is dispensed exclusively at community pharmacies, while others have a mix of community pharmacy and public clinics. New South Wales has the majority of public OAT clinics in the country but patients must attend community pharmacies if they want to access unsupervised dosing (takeaways) [11]. Nationwide, the majority of dosing points (89%) are located in pharmacies [11]. Community pharmacies offer longer opening hours and more accessible locations than public clinics, yet the out-of-pocket dispensing fees at pharmacy can make OAT prohibitively expensive [12].There are 2673 OAT prescribers of which 83% work in the private sector, such as private general practice [11].

Towards the end of the first year of the COVID-19 pandemic, at the end of 2020, Australia was maintaining a rate of around 0.5 cases per million people per day while countries in Europe experienced new peaks (371 and 580 cases per million people per day in the UK and Italy, respectively) [13]. Within Australia, states and jurisdictions mandated restrictions: the city of Melbourne was in lockdown from July to October 2020, while Adelaide had a lockdown of 6 days in November 2020 [14]. In spite of the comparatively low case numbers, measures to prevent COVID-19 transmission in the healthcare setting were put in place across Australia.

Studies investigating increased flexibilities in OAT provision during the COVID-19 pandemic period have not found an associated increase in opioid overdose [15–17]; therefore these flexibilities could be a catalyst for long-term change in the system. Understanding the mechanisms which facilitated these flexibilities will help identify pathways to sustained change.

Deimplementation is the “removal or reduction of costly or potentially hazardous approaches to care” [18], aiming to improve public health [19]. Deimplementation is distinct from implementation, in part because the work involved is contingent on the complexity of the intervention already in place [19]. While prior literature argues the merits of deimplementing healthcare *overuse* to achieve equity, such as reducing use of preventive care and screening which delivers no benefits [20], less attention has been paid to deimplementation of burdensome healthcare or healthcare involving intrusive surveillance that creates or perpetuates inequity. In relation to opioids and OAT, deimplementation has historically been carried out as reductions in prescribing rates [21] yet evidence indicates that this decreased access to prescribed opioids may increase harms by increasing use of unregulated, illicit drugs [22, 23]. Assessments of psychosocial stability and comorbidity have not been found to have a clear association with patients’ adherence to treatment [24] and assessments based on arbitrary criteria may exacerbate social inequities in health by allowing prejudices to be perpetuated. Deimplementation of practices including removal of supervised dosing, urine drug screening, and frequent in-person attendance for review, could move services towards more equitable, patient-centred OAT provision.

Inadequate access to health services is an important determinant of social inequity in health. Social inequities in health are “differences in health that are avoidable and also considered unfair or unjust” [25]. For people who inject drugs, previous studies have found characteristics such as gender, rurality, and ethnicity to be associated with inequitable retention in OAT [26]. Health services which ignore issues of geographic, economic, and cultural access to healthcare [27], risk perpetuating social inequities in health. A person’s built environment, social environment, and the healthcare infrastructure can condition ability to negotiate complex health services [28], amplifying the importance of simplified pathways in care. The restrictions prevalent in OAT programs work in tandem with societal inequalities to produce inequitable access to healthcare, resulting in potentially harmful events such as premature discharge from OAT programs [29]. On-demand, flexible, and destigmatising drug treatment has been proposed as a way to deliver equitable care in an unequal society [30]. Literature on health equity in implementation has focused on context and system factors while less attention has been paid to the clinical encounter [31]. The numerous competing priorities within health services mean an equity lens is not always integrated in the planning stage of an intervention [32], especially in times of rapid change such as the COVID-19 pandemic. Although equity was not embedded in changes to OAT services, using an equity lens to investigate the work of providers and their clinical encounters during the COVID-19 pandemic can generate evidence to sustain deimplementation.

Normalisation Process Theory has been employed extensively in primary care settings [33] and, to a lesser extent, in healthcare interventions related to substance use [34] to understand and evaluate processes of “adoption, implementation, and sustainment of socio-technical and organisational innovations” [35]. Normalisation Process Theory is pertinent to the systems of OAT provision which have long been considered complex because of their strict regulatory oversight and the varied interpretation of guidelines among OAT prescribers [36]. The theory extends the understanding of complex healthcare interventions by investigating an intervention as an assemblage of beliefs, behaviours and practices, whose outcomes are contingent on its context [35]. Normalisation Process Theory has previously been employed to investigate the reduction of low-value healthcare, predominantly around prescribing practices, i.e. reducing inappropriate antibiotic use [37]. Using concepts from Normalising Process Theory, this analysis investigated how providers considered social inequity in health of patients in the deimplementation of restrictive OAT provision during the COVID-19 pandemic.

## Methods

The CHOICE Study aimed to evaluate the impact of the COVID-19 pandemic and related restrictions on the delivery of drug treatment services in Australia from the perspectives of people receiving and providing OAT [38, 39]. Semi-structured interviews were completed between August and December 2020 via telephone and videocall. People receiving OAT were recruited via study dissemination through members of the community reference panel in their capacity as staff in eight peer-led organisations in seven Australian jurisdictions. Doctors, nurses, and service managers who provide OAT were recruited via a study flyer disseminated through existing research networks and additional providers were recruited by study flyer to staff at two drug treatment clinics. The sample was recruited to reflect a range of jurisdictions.

All participants were reimbursed AUD$50 cash transfer or gift voucher (according to their preference) for their time and expertise. Participants provided verbal consent prior to the interview. Audio recordings of the interviews were transcribed verbatim by a transcriber working under a confidentiality agreement. Transcripts were deidentified and checked for accuracy by AC. Participant numbers are provided but no other further information to avoid inadvertent identification.

This analysis draws on the interviews carried out with OAT providers in the CHOICE Study. We employed an abductive approach, beginning with careful methodological analysis to identify unexpected findings in the data and inform an explanatory hypothesis [40]. This was done by clustering codes related to the determinants of retention in OAT to explore variation in providers’ descriptions of the relationship between deimplementation and social inequities in health. The determinants of retention in OAT were identified in prior literature: sex [41], age [42, 43], arrest/incarceration history [41, 43], comorbidities [42], stigma [44], and regulations governing OAT [45]. Analysis of the resulting code clusters engages with constructs from Normalising Process Theory [35] (1) Adaptive execution: How do contexts affect the ways in which OAT service staff can adapt to make an intervention and its components a workable proposition in practice? (2) Cognitive participation: How do prescribers and colleagues work together to create networks of participation and communities of practice around interventions and their components? (3) Normative restructuring: How has working with interventions and their components changed the norms, rules and resources that govern how people work?, and (4) Sustainment: How have interventions and their components become incorporated in practice?. This allows an exploration of how providers understood their work during the COVID-19 pandemic as explicitly or implicitly responding to structural determinants which conditioned OAT access. AC led the coding, data summary and analysis processes and the research team met regularly to review developing interpretations, including drawing on co-authors’ expertise of receiving (SC) and providing (JH) OAT.

Consistent with other countries [17, 46–48], OAT services in Australia adapted to reduce possibilities of COVID-19 transmission. For our analysis, deimplementation refers to changes described in the “Interim guidance for the delivery of medication assisted treatment of opioid dependence in response to COVID-19: a national response” [49], including reducing supervised dosing, reducing in-person appointments (via initiation onto depot buprenorphine or transfer to telehealth), and reducing biological monitoring such as urine drug screening.

## Results

This analysis explores variation in how OAT providers relate their work in deimplementation to concepts of social inequity in health during the COVID-19 pandemic. Interviews were conducted with 29 OAT providers in New South Wales, Victoria, Queensland, Western Australia and Australian Capital Territory (59% doctors, 31% nurses, 10% service managers; average number of years working in OAT was 11).

### Adaptive execution: how do contexts affect the ways in which OAT service staff can adapt to make an intervention and its components a workable proposition in practice?

Providers suggested that “one size fits all” deimplementation would be harmful to some patients, and adaptive execution was evident in several accounts. Such adaptive execution allowed providers to tailor the deimplementation to what they perceived were the needs of subpopulations. P01 described overcoming the challenges of reducing in-person appointments for people for whom telehealth was not appropriate. The adaptive execution of deimplementation allowed more equitable care to be provided to people with comorbidities.

P01: The number of people who can’t manage telephone consultations—don’t have access to a telephone—is much smaller but there’s a cohort of clients who are homeless or have significant psychiatric comorbidities like schizophrenia or intellectual disability. So, those who tend not to come into appointments in general and [don’t] turn up in the right week at the right time for their appointments. Who also don’t have a telephone. So there’s some clients that need sort of fairly flexible care who we’re seeing in person, essentially, when they turn up.

For people who were isolating with a COVID-19 diagnosis or isolating because of comorbidities which increased vulnerability to COVID-19 transmission, home delivery was offered at some sites. P08 described the adaptive execution when home delivery was not feasible, through flexible OAT pick-up practices to ensure continuity of care.

P08: We would never have been in a position where we had anywhere near the staffing capacity to actually [deliver OAT to homes]. Plus, we felt that the alternative, which was get mum or uncle, or brother, or somebody to go and pick it up for you, we felt that was an appropriate way around that.

Accounts of adaptive execution demonstrated the tensions between providers’ treatment goals and the equitable provision of care, particularly when other services were being provided alongside OAT. P21 reported that in-person reviews allowed for people with “mild or moderate psychotic symptoms… to be scheduled or encouraged to voluntarily go into the mental health unit”. In P20’s clinic, increased flexibilities in OAT provision were withheld to achieve hepatitis C treatment completion.

P20: Sometimes patients who are having hepatitis treatment provided at the clinic were kept a bit longer [on supervised dosing] as well, to make sure that they could continue the hepatitis treatment, knowing that some of those patients weren’t taking their dose, if they weren’t at the clinic, being supervised and helped by taking that. So … I think they were the primary patients that were kept at the clinic.

One nurse felt the workarounds risked damaging patient-practitioner relationships. The different treatment of patients which is inherent in equitable care was seen as problematic when treating a relatively closed community of people attending the service.

P14: I understand why [the service] had to stick to generalised rules for everyone because otherwise the lines get a bit grey. I sometimes wish [the rules] were a bit more black and white because you do find, looking after 60 clients, one person will say, “Well, how come Dave got this?” And you’re like, “Well, because of the situation.” They’re like, “That doesn’t feel a bit fair.”

### Cognitive participation: how do prescribers and colleagues work together to create networks of participation and communities of practice around interventions and their components?

The entrenched rigidity of OAT restrictions in Australia was a challenge to deimplementation. Communities of practice to support deimplementation, were more easily formed when providers within a service deemed deimplementation to be coherent with the existing organisation ethos. This sense-making process was most visible where providers situated their work within the principles of harm reduction, indicating the service’s prioritisation of equitable provision of care over abstinence and treatment cessation.

P15: I mean, if someone had had a one-off use of [methamphetamine] and had told you about it, that’s not really going to affect the take-away status. So you’ve got to look at things in their entirety. And [our service] is a harm minimisation program. We don’t promise a cure. So some people will use occasionally and that’s a reality. But the program has helped them limit their use and improve their life, so you’ve got to look at all things, not just one thing in isolation.

Where deimplementation was carried out without evident support from a community of practice, the providers expressed confidence in their own decision-making. Although P03 acknowledged the limits of the setting (public clinic vs private), they also felt assured in grounding their decisions in “clinical rationale”.

P03: As a public clinic, we probably always err on the side of caution with takeaways and we’re much, much more likely to stick to the state guidelines than many of the private prescribers. But I think as long as you’ve got a good clinical rationale for what you’re doing we can really sort of push for ourselves to be a little bit more flexible. So, in that regard, it’s a real positive I think.

In some instances, the common experience of the COVID-19 pandemic encouraged the establishment of a community of practice, which some providers used to mobilise efforts towards sustained deimplementation. Communities of practice are an opportunity for the clinicians who are driving deimplementation to support others who are lagging behind. P23 reported using the period of rapid change to gain the support of hospital staff, by providing education on the benefits of flexible OAT provision based on their own experience. Providers who extolled deimplementation to achieve social equity in health were previously advocating to individual clinicians but found that COVID-19 had broadened interest to a wider community of clinicians. For P27, who had long been advocating for more flexibility in OAT provision, deimplementation during COVID-19 was an opportunity to change from incremental change to a more drastic shift towards “humane” care that P27 had long supported.

P27: I had my sort of resistance movement in the corner just to the extent that I could as a [senior staff member]. I write my own rule book. You’d convince a few people along the way that this was better medicine and more humane whereas now you’ve got an axe that’s gone right through the whole thing, and everybody realises, “Oh, you can give people all these take-aways and you don’t have a rash of overdoses. And we may be even improving the outcomes.”

There was a recognised need for communities of practice which incorporated health services beyond drug treatment, but these were stymied by siloed healthcare systems. P08 felt deimplementation processes could be smoothed through collaboration between addiction medicine and mental health. Consistent with prior literature [50, 51], breaking down siloed healthcare was deemed to be an important facet of delivering equitable care that addresses multiple needs beyond OAT. The belief that OAT was not part of core business in mental healthcare was identified as a barrier to optimising implementation.

P08: Mental health services are set up quite nicely around the regions [and there is potential for them to deliver] depot buprenorphine which we think will be a simpler way of delivering pharmacotherapy, but people aren’t terribly interested. The response, generally, is, “Look, we’re really busy. We’re just not going to take that on”.

### Normative restructuring: how has working with interventions and their components changed the norms, rules and resources that govern how people work?

After decades of OAT provision with little innovation or change, the normative restructuring required for deimplementation is a key area to address in the normalisation process. The work involved in deimplementation for the COVID-19 period provoked reflection on the norms around OAT provision and caused providers to question their own motivations as well as wider treatment motivations. The tasks that were involved in deimplementation challenged P30 to consider their identity as a health worker, despite being initially scared of an increased threat of COVID-19 and changes to the handling of methadone and buprenorphine.

P30: For example, home dosing [could be a possibility] not just for things like COVID but for people that are maybe very, very ill or terminally ill, you know, that can now be [considered]. Initially, as a health professional, I was very scared. So, if I’m very honest, home dosing wasn’t something that I wanted to do, initially. I have pre-existing conditions myself so [I was thinking about] whether […] I would be more impacted if I got infected [with COVID]. And also […] we are delivering an S8 drug [drugs that are subject to tight restrictions] and sometimes to homes that are in suburbs or areas that are not very safe.

Strategies to meet the needs of certain groups of patients included forming communities of practice across other services or with clinic staff who were not OAT providers. P13 described their service’s successes in “bridging the gap” to more equitable care for Aboriginal people through sessions at an Indigenous Health Service. P26 highlighted the increased importance of case managers during the COVID-19 pandemic which improved care coordination but also supported the transition from a “medical”, “pharmacotherapy-based service” to one which could support people’s broader needs. Although there is no standard definition of a case manager (it does not preclude being an OAT provider), they broadly work with the patient on issues beyond treatment which may impact a person’s ability to engage in OAT.

P26: The caseworkers, you know, nurses, social workers, psychologists, are probably a bit more instrumental in delivering care or being responsible for organising and co-ordinating care. I think previously, because we’ve got a predominantly pharmacotherapy-based service, it became quite medical. And there was [previously] a lot of opportunity for clients to circumvent their case-management processes because, really, all they want is a script. So, if all I want is a script, all I need to do is go and see the doctor for my script review. So there’s been a slight shift in focus towards case management.

While community pharmacies constitute the majority of dosing points in Australia, the COVID-19 pandemic saw their increased use in OAT delivery as OAT clinics moved people to pharmacy in order to receive takeaways. P18 indicates normative restructuring was required among people receiving OAT, to allow pharmacies to perform this role in deimplementation. P18 highlights that established relationships with a provider in the clinic could make people receiving OAT, who often experience stigma, reticent to attend pharmacy. Ensuring that all health services, including pharmacy, provide non-stigmatising care is paramount to achieving deimplementation and equitable care.

P18: Many of our patients experience stigma and discrimination but sometimes they fear stigma and discrimination. And, and I think a lot of them found that, when they did go to the pharmacy, the stigma and discrimination that they might have been expecting didn’t, in fact, occur or occurred to a lesser degree. And, you know, the, the comfort that they had with our nurses was then transferred into comfort that they had with their community pharmacies. And so, having sort of made the leap, they then realised that some of the fears that they had about it previously were not so founded and became comfortable in their new environment.

Some norms relating to OAT restrictiveness remained unchallenged and could present a barrier to deimplementation. Drug treatment is notable because the evaluation of a patient’s success based on “good behaviour” or compliance with an explicit or implicit set of rules. P23 reported that in-person attendance was necessary to assess if the person was “drowsy”, “dishevelled’, or “well-kempt”, underscoring the breadth of criteria beyond the official guidelines which providers apply when assessing a patient’s engagement in treatment. P22 reported that looking for signs of ongoing drug use such as “hobbling in because they have an infection in their groin”, was part of a risk management strategy and stated that using “that leverage to bring them back in [for more frequent in-person dosing] is all about safety; nothing else”. These criteria based on appearance, of which the person receiving treatment may not be aware of, creates a “black box” of clinical decision-making and prevents people engaged in treatment from advocating for their own care. The tacit criteria described above highlight the many ways by which a provider might consider somebody “unsuccessful” in treatment. Basing treatment decision-making on superficial or non-clinical indicators, without first discussing needs and a corresponding treatment plan with the person who is in treatment, risks exacerbating the social and structural determinants of poor health.

### Sustainment (normalisation): how have interventions and their components become incorporated in practice?

Despite the study taking place during the first year of the COVID-19 pandemic, providers were already beginning to reflect on the service in a “post-COVID-19 world” and they highlighted several barriers to normalisation. There was a sense that reduced in-person appointments were not suitable for everyone and would inherently result in inequities in provision of care. P02 felt that the service was not equipped to offer telehealth to people who had lower levels of English, excluding them from more flexible care.

P02: [I would recommend that people attend in person if] their English language skills aren’t that good. That’s nearly impossible to do on the phone or by telehealth. So, I ask them to come in because I think anything on the phone would be so cursory and superficial it would be a waste of time. At least face-to-face when there’s a language barrier… I can use Google Translate and there’s all sorts of things I can do which I can’t do otherwise.

Providers were concerned that COVID-19 exceptionalism would not last long and any adverse events (such as overdose) that might be attributed to increased access to OAT takeaways could provoke legal consequences. In P16’s view, this limited the possibilities for sustained deimplementation, despite being generally supportive of the process during the pandemic.

P16: I like to be able to sit down with the patient and negotiate an acceptable number of take-aways based on their current situation, and what I perceive to be the safe, safeness of the situation according to the guidelines. But I don’t feel that a formal documentation helps me particularly… I’d like to go back to the more restrictive practice mainly to protect myself more than anything else. From the medico-legal point of view.

P11 highlighted the ongoing issue that jurisdictions differ in their OAT guidelines and regulations. P11 calls for different types of evidence to be drawn upon in order to shape sustained deimplementation, suggesting that “expert consensus” could play a key role given the perceived lack of other evidence.

P11: My simple understanding of that is that, when there’s that sort of disparity between two jurisdictions for something that is medically supervised, the evidence probably is limited as to what works and what doesn’t work. Otherwise, we’d be following whatever evidence was available, I would have thought. And, in the absence of evidence, my understanding is that the next step to look at in terms of guidance about an issue in medicine is expert consensus. The difference is quite marked in terms of supervision of OAT between those two jurisdictions [in Australia].

## Discussion

By investigating change in the provision of OAT during COVID-19, this analysis from the CHOICE Study provides useful insights for future work around deimplementation and equity beyond the OAT setting. Deimplementation in OAT provision presents an opportunity to reduce burden of treatment for patients, reduce resource outlay in clinics, and improve patient and provider satisfaction. Deimplementation which prioritises equity in health and maximises efficient use of resources [19] is vital, particularly following the COVID-19 pandemic in a landscape of increasing pressure on health services in terms of finances and staffing. This analysis demonstrates that the constructs from Normalisation Process Theory can advance understandings of social equity in health in the work that is done by OAT providers in processes of deimplementation.

The typical decision-making processes of healthcare providers were disrupted by the pandemic, where the quickly evolving situation challenged evidence-based medicine paradigms [52]. Providers who understood the need for responsive healthcare enacted an “evidence-enough” approach situated in the local context [53]. Nevertheless, when considering normalisation, providers expressed fears and assumptions about legal repercussions for themselves which outweighed the need for treatment flexibility. Providers sought to inform decisions with specific types of evidence ranging from expert consensus to jurisdiction-level monitoring of overdose. In other countries, investigations of the impact of increased flexibilities in OAT provision during COVID-19 have so far found no evidence of an associated increase in opioid-related mortality [15–17, 54]. Limited notions of evidence proposed by OAT providers in our analysis left little room to incorporate the experiences or metrics deemed important to the people that use the service. Prior research from this study has highlighted the need to evaluate services through a wider set of measures, beyond just medication-related outcomes [39]. By primarily focusing on jurisdiction-level monitoring of overdose to inform decisions on OAT provision, providers create a hierarchy of evidence, detracting from other patient-centred measures of treatment success such as shared decision-making and individualised care [55]. The hierarchy is not conducive to developing nuanced understandings of the treatment experience which are necessary to cultivate change [56]. While some people receiving OAT value reduced in-person attendance because it allows for feelings of “normality” and flexibility in daily life patterns [8], these are outcomes rarely reflected in evaluation of OAT services. To achieve equitable health outcomes for people receiving OAT, discussions with patients about treatment goals to establish treatment provision must become the norm. The co-creation of indicators to monitor OAT services is an important step to ensuring relevant outcomes are being measured and equitable health is being achieved. Existing research on patient-centred outcomes provides a baseline for key indicators of interest at the service level (e.g. provision of empathetic and non-judgemental care, shared decision-making [55]) and patient level (e.g. avoidance of withdrawal, improvement of familial/social relationships [57]). Integrating these indicators into OAT guidelines with the goal of improving the patient experience could support the normative restructuring needed within the service to sustain change.

To reduce social inequities in health, deimplementation needs appropriate, non-stigmatising healthcare outside of the OAT setting. One participant noted that people may be reticent to change dosing point, supporting literature on potential stigma produced through negotiating treatment in unfamiliar territory [58]. There is potential in delivering other health services in the drug treatment setting [51], but this should not negate the rights of people who use drugs to use mainstream health services without discrimination. Several providers saw a key role for mental health services in deimplementation but noted that addiction and mental health remain siloed in many regions, as previously reported in Australia [59]. Deimplementation could make space for drug treatment clinics to become “drug user health hubs”, which integrate voluntary care for hepatitis C treatment, harm reduction services, and culturally competent mental health treatment [60]. Centring patient choice in decisions about how and where people receive healthcare is fundamental to the success of deimplementation in the drug treatment setting [8], both for OAT and other healthcare frequently utilised by people who use opioids. Collaboration across health services could improve OAT access as well as complement the rollback of frequent in-person reviews which patients may use to address wider health concerns.

There were concerns raised about the unequal treatment of patients, which is inherent to equitable healthcare, and its impact on patient-practitioner relationships. Community dynamics give people the information and tools to advocate for their own access to OAT using the knowledge and experiences shared with peers. This may benefit people who have stronger social networks or are linked to community organisations, possibly producing inequities with people outside of these networks. While there is evidence on the importance of social networks in OAT outcomes [61], there is less investigation on the influence of social networks on treatment encounters. Groups that may not benefit from those social networks include people from culturally and linguistically diverse backgrounds. There are few studies on treatment practices for people from culturally and linguistically diverse backgrounds [62], and our analysis demonstrates people who do not speak English as their first language could be excluded from changes to OAT services when providers assume they are unsuitable candidates for telehealth. A participant also suggested that flexibilities were more readily available for people attending private clinics over public, which could reproduce social inequities in health caused by income disparities. Additionally, although people in rural areas would be primary beneficiaries of deimplementation [63], they may be less connected to peer-led organisations which may limit their involvement in patient advocacy for deimplementation. In several jurisdictions in Australia, peer-led organisations lead groups which provide helplines with support and advocacy for people having issues in their opioid treatment program. These organisations are well-positioned to report issues of treatment access for people in regional areas and improve equitable deimplementation.

Normalisation of deimplementation requires “thought leaders” and communities of practice to disrupt self-identities of prescribers who are more reticent to adopt. Diffusion of innovation theory [64] has been used to investigate drug treatment practitioner experiences of providing hepatitis C treatment [50] and is helpful to explore how different groups have different needs to shift perspective and enact change. Some participants indicated that doing the work required for deimplementation had changed their own professional identity, showing promise for providers being willing to adapt to sustain deimplementation [65]. Services varied in the extent to which staff were united in driving forward deimplementation, but this revealed the importance of those few key “thought leaders” who report advocating for flexibilities in OAT provision for many years. Linking practice to providers’ peer and reference group behaviours can support sustained deimplementation [66]. More research is needed to understand how the “thought leaders” have impact within different clinic structures, i.e. understanding if prescribers influence case managers in the same way they influence other prescribers.

There are several limitations to the study. The initial OAT providers who participated in the study were contacted because they currently or previously participated in research, which could result in a sample already interested in patient-centred care. Participants were recruited via public clinics which limited the opportunities of reaching providers working in the private sector. There were two jurisdictions (Northern Territory and Australian Capital Territory) where providers were not recruited. The interviews took place over a period of four months at the end of 2020 and given the rapidly changing COVID-19 case numbers, restrictions and differences in state response, the time of enrolment and location of participant may have influenced participants’ responses.

The findings from this analysis indicate a number of key areas for policy change. Deimplementation has the potential to improve equitable outcomes in health, but only if the patients’ treatment goals are centred in the provision of OAT without compromising access to flexible care. Discussions with the person receiving OAT about their treatment goals should inform treatment initiation and any ongoing reviews of treatment. Models of “open access”, which prioritise the removal of barriers to care to ensure rapid initiation onto OAT, can help guide service planning [67]. Globally, there are many examples of peer-led harm reduction and hepatitis C services [68, 69] yet few examples of peer-led OAT services. Involving peer-based organisations in the design and delivery of OAT programs can help compensate for inequities that are being produced by the clinical encounter. Deimplementation will reduce contact with an OAT program for the majority of people, so it is increasingly important that other health services are providing non-stigmatising care to people receiving OAT. Clinicians can be supported to equitably implement deimplementation with support from “thought leaders”. Clinicians with experience delivering healthcare in peer-led organisations likely have the appropriate experience to support colleagues.

## Conclusions

In this account of deimplementation, providers linked social equity in health to their work despite equity not being embedded in the planning stage. Provider norms and OAT regulations prevent an equitable, flexible, system of OAT provision from being actualised. The flexibilities introduced during COVID-19 provoked OAT providers to consider the cost of overly restrictive treatment programs. The providers reflected on their understanding of the balance between hazard and safety, in the context of peripheral threats to health and wellbeing generated from restrictive OAT provision. This analysis demonstrates the importance of centring social equity in health in the planning of deimplementation strategies. Normalisation Process Theory is useful to explore potential problems in the deimplementation process which could prevent normalisation.

## Data Availability

The datasets generated and/or analysed during the current study are not publicly available due to protect the identity of participants.
